# Focus Group Study of Medical Stakeholders to Inform the Development of Resilient Together for Dementia: Protocol for a Postdiagnosis Live Video Dyadic Resiliency Intervention

**DOI:** 10.2196/45533

**Published:** 2023-05-29

**Authors:** Sarah Bannon, Julie Brewer, Talea Cornelius, Jonathan Jackson, Robert A Parker, Kristen Dams-O'Connor, Bradford Dickerson, Christine Ritchie, Ana-Maria Vranceanu

**Affiliations:** 1 Center for Health Outcomes and Interdisciplinary Research Department of Psychiatry Massachusetts General Hospital Boston, MA United States; 2 Harvard Medical School Boston, MA United States; 3 Center for Behavioral Cardiovascular Health Columbia University Irving Medical Center New York, NY United States; 4 Community Access, Recruitment, and Engagement Center Division of Clinical Research Massachusetts General Hospital Boston, MA United States; 5 Biostatistics Center Massachusetts General Hospital Boston, MA United States; 6 Brain Injury Research Center Department of Rehabilitation and Human Performance Icahn School of Medicine at Mount Sinai Hospital New York, NY United States; 7 Department of Neurology Icahn School of Medicine at Mount Sinai Hospital New York, NY United States; 8 Frontotemporal Disorders Unit Departments of Neurology Massachusetts General Hospital Boston, MA United States; 9 Department of Psychiatry Massachusetts General Hospital Boston, MA United States; 10 Mongan Institute for Aging and Serious Illness Department of Medicine Massachusetts General Hospital Boston, MA United States; 11 Division of Palliative Care and Geriatric Medicine Department of Medicine Massachusetts General Hospital Boston, MA United States

**Keywords:** dementia, stakeholders, focus group, intervention, video, telehealth

## Abstract

**Background:**

Alzheimer disease and related dementias (ADRD) are increasingly common conditions that disrupt the lives of persons living with dementia and their spousal care partners. At the time of ADRD diagnoses, many couples experience challenges that produce emotional distress and relationship strain. At present, there are no interventions to address these challenges early after diagnoses to promote positive adjustment.

**Objective:**

The study protocol described here is part of the first phase of a larger program of research that aims to develop, adapt, and establish the feasibility of Resilient Together for Dementia (RT-ADRD), a novel dyadic skills-based intervention to be delivered over live video early after diagnosis, with the goal of preventing chronic emotional distress. This study will elicit and systematically summarize perspectives of ADRD medical stakeholders to inform the procedures (eg, recruitment and screening methods, eligibility, timing of intervention, and intervention delivery) of the first iteration of RT-ADRD prior to pilot-testing.

**Methods:**

We will recruit interdisciplinary medical stakeholders (eg, neurologists, social workers, neuropsychologists, care coordinators, and speech language pathologists) from academic medical center clinics in the departments treating persons living with dementia such as neurology, psychiatry, and geriatric medicine via flyers and word-of-mouth referrals from clinic directors and members of relevant organizations (eg, dementia care collaboratives and Alzheimer disease research centers). The participants will complete electronic screening and consent procedures. Consenting individuals will then participate in a 30- to 60-minute qualitative virtual focus group, held either via telephone or Zoom, using an interview guide designed to assess provider experiences with postdiagnosis clinical care and to gather feedback on the proposed RT-ADRD protocol. The participants will also have the opportunity to participate in an optional exit interview and web-based survey to gather additional feedback. Qualitative data will be analyzed using a hybrid inductive-deductive approach and the framework method for thematic synthesis. We will conduct approximately 6 focus groups with 4-6 individuals in each group (maximum N=30 individuals; until saturation is reached).

**Results:**

Data collection began in November 2022 and will continue through June 2023. We anticipate that the study will be completed by late 2023.

**Conclusions:**

The results from this study will inform the procedures of the first live video RT-ADRD dyadic resiliency intervention focused on the prevention of chronic emotional and relational distress in couples shortly after ADRD diagnoses. Our study will allow us to gather comprehensive information from stakeholders on ways to best deliver our early prevention–focused intervention and gain detailed feedback on study procedures prior to further testing.

**International Registered Report Identifier (IRRID):**

DERR1-10.2196/45533

## Introduction

### Background

Alzheimer disease and related dementias (ADRD) are a common cause of death and disability that have a substantial impact on individuals, families, health care systems, and society. By 2030, over 78 million individuals will be living with ADRD, leading to global health care costs of over US $2.8 trillion [1,2]. There are no available disease-modifying treatments or cures for ADRD [2,3]. ADRD symptoms (eg, forgetfulness, communication challenges, loss of recognition of places, time, and routines) are progressive in nature and continually undermine the independence of persons living with dementia [2]. In the months and years following diagnosis, these symptoms lead to heightened stress, clinically elevated emotional distress, burden, and relationship strain for persons living with dementia and their family care partners—many of whom are spouses or other forms of committed partners [4-7]. The timely diagnosis, care, and management of ADRD is critically important because these can each facilitate positive adjustment and preserve quality of life for couples [8,9].

Recent research advancements have enabled the earlier diagnosis of ADRD, creating the potential to meaningfully engage persons living with dementia in psychosocial interventions early after diagnosis during the “window of opportunity” before cognitive symptoms progress [9]. However, there are no established psychosocial interventions available at the time of diagnosis for persons living with dementia and their partners to support them in developing a shared understanding of the diagnosis, learning to collaboratively address early challenges, expressing personal needs, and collaboratively planning for the future [10,11]. Evidence suggests persons living with dementia and partners are interested in participating in interventions together (ie, “dyadic interventions”), early after diagnosis [10-14], and prior work has demonstrated that dyadic interventions are more effective avenues for promoting positive adjustment than those focused on individuals or care partners alone [15,16]. Dyadic interventions deployed during the later stages of ADRD have demonstrated feasibility and some positive effects on the dyads’ relationships [17], including those delivered via telehealth approaches (eg, Zoom; Zoom Video Communications) [18-20].

Early dyadic interventions have the potential to promote positive adjustment to ADRD by improving the dyads’ individual and interpersonal *resiliency* (ie, the ability to adjust effectively to significant adversity) [21] and can provide persons living with dementia and their partners with skills and support to actively contribute to care planning, preserve autonomy for persons living with dementia, and help both partners to express their needs and preferences [22,23]. The members of our team have developed dyadic programs with procedures that can be adapted to address the dyads’ needs early after ADRD diagnoses. Specifically, the Recovering Together program is a brief dyadic resiliency intervention that demonstrates feasibility and preliminary efficacy at preventing chronic emotional distress in patients and informal care partners (ie, unpaid family members and friends) shortly after intensive care unit admission for acute neurological illnesses (eg, traumatic brain injury and stroke) [24,25]. The Recovering Together program was iteratively developed as part of a larger multidisciplinary partnership with patient–care partner dyads and medical stakeholders involved in patient care [26]. Prior research indicates that psychosocial interventions for ADRD are poorly integrated into medical treatment settings, have inconsistent methods of delivery, and are poorly aligned with participant and medical stakeholder preferences [14,27]. Therefore, there is a missed opportunity to integrate dyadic psychosocial interventions into the postdiagnosis support clinical infrastructure, and to design interventions that consider the perspectives of participants and medical stakeholders involved in patient care. Gathering feedback from individuals who will adopt, deliver, and use interventions is a necessary first step of developing early psychosocial interventions to improve adjustment to ADRD diagnoses for persons living with dementia and their care partners [28,29].

### This Study

**Figure 1:**
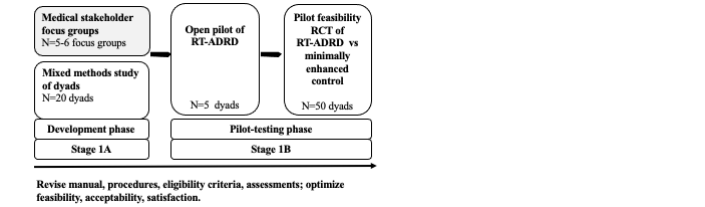
Proposed iterative development of Resilient Together for Dementia (RT-ADRD). RCT: randomized controlled trial. RT-ADRD: Resilient Together for Dementia.

The purpose of this paper is to describe the protocol for our qualitative focus group study involving ADRD medical stakeholders that will inform the development of the procedures of the first version of a dyadic intervention. The study protocol described here is part of the first phase of a larger 5-year program of research (Figure 1) that aims to use the methodology, program content, and procedures of the Recovering Together program as a basis for developing a novel postdiagnosis dyadic resiliency intervention for ADRD called Resilient Together for Dementia (RT-ADRD). RT-ADRD will be developed using our team’s theoretically and empirically derived framework for transdiagnostic dyadic interventions [30]. Our approach to intervention development will follow the National Institutes of Health stage model for intervention development [31], including the following: (1a) qualitative interviews with dyads and (1b) qualitative focus groups with ADRD medical stakeholders to understand population needs and develop the first iteration of the program; (2) an open pilot study to assess and refine RT-ADRD content and procedures; and (3) a feasibility pilot randomized controlled trial to further refine RT-ADRD content and test feasibility, acceptability, and preliminary efficacy of RT-ADRD relative to a minimally enhanced usual care condition (Figure 1). Through this study, we will gather detailed information from heterogeneous groups of medical stakeholders and develop procedures (eg, recruitment, screening, eligibility, timing of intervention, and intervention delivery) that are feasible, acceptable, and capable of meaningfully improving adjustment to ADRD for persons living with dementia and their partners.

## Methods

### Ethics Approval

This study was approved by the Massachusetts General Independent Review Board (2022P001510).

### Study Design

We will conduct a qualitative focus group study involving medical stakeholders (eg, neurologists, geriatricians, geriatric psychiatrists, social workers, neuropsychologists, care coordinators, and cohabiting speech language pathologists) involved in the care of persons living with dementia around the time of diagnosis (target N=up to 30 stakeholders in up to 6 focus groups). This study is designed to gather ADRD stakeholders' perspectives on the following: (1) postdiagnosis challenges and psychosocial support needs of persons living with dementia and their partners, (2) factors that may promote or inhibit couples’ suitability and interest in a postdiagnosis dyadic intervention, and (3) proposed recruitment procedures and ways of optimizing recruitment support from ADRD stakeholders.

### Inclusion and Exclusion Criteria

We plan to include up to N=30 adult participants (18+ years old) who are currently employed in 1 of 2 academic health care settings and provide outpatient care for individuals diagnosed with ADRD around the time of diagnoses. Recruitment began in November 2022 and will continue until June 2023. Eligible participants will be (1) fluent in English, (2) willing and able to participate in a live video interview or focus group, and (3) employed in a position relevant to ADRD care. Focus groups will be conducted separately for each of the 2 recruitment sites in order to understand potential differences by clinic setting. Our team will use purposive, convenience sampling from both sites and construct focus groups in an iterative manner based on ongoing data collection and rapid data analysis, consistent with prior studies [32].

### Recruitment and Screening

We will recruit participants actively involved in ADRD clinical care from clinics within relevant departments (eg, neurology, psychiatry, and geriatric medicine) at 2 academic medical centers with comprehensive research and clinical programs dedicated to the treatment of ADRD (Massachusetts General Hospital in Boston, MA and Mount Sinai Hospital in New York, NY). We will recruit via flyers, presentations, and word-of-mouth referrals. Interested individuals will respond to a brief survey on a secure Research Electronic Data Capture platform and provide their name, contact information (email or phone number), current employment site, and current position. The survey will also contain an information sheet and form for eligible individuals to provide electronic consent to participate in the study. A research assistant will contact the individual to confirm eligibility and discuss scheduling for a focus group or individual interview. All recruitment, screening, and consent procedures will be performed remotely via telephone, electronic survey, email, and over a live video platform (ie, Zoom).

### Data Collection

We will conduct a series of focus groups comprising medical stakeholders. Focus groups will include approximately 4-6 participants to encourage discussion while maximizing participant comfort, consistent with guidelines for virtual focus groups [33]. Stakeholders who are unable to attend the focus group sessions will be invited to participate in an individual interview. Focus groups and individual interviews will last approximately 30-60 minutes in total. Focus group participants will have the opportunity to participate in an optional exit interview immediately following the conclusion of the focus group to provide any additional information that they did not have time to share or did not feel comfortable sharing in the group setting.

Focus groups will be cofacilitated by a PhD-level clinical psychologist with expertise in qualitative research methods and a trained research assistant. The clinical psychologist will lead the focus group, and the research assistant will provide technical support and document observations using a rapid data analysis template developed by the research team. The rapid data analysis template will be used to inform formal coding and includes observations within key interview domains, poignant participant quotes, researcher reflexivity, and other notes or observations (Multimedia Appendix 1) The clinical psychologist will work to generate information on convergent and divergent perspectives regarding the dyads’ experiences early after ADRD diagnoses and feedback surrounding the proposed intervention procedures and content. Exit interviews will be led by trained research assistants and volunteers using a live video conference breakout room tool.

Prior to the focus groups, participants will receive a document with potential discussion topics and guidelines for participating in the focus group with other individuals. The focus group discussion will follow an interview guide with open-ended prompts and questions surrounding domains including the following: (1) perceptions of clinical care early after ADRD diagnosis, (2) feedback on the proposed intervention procedures and content, and (3) ways of maximizing feasibility and acceptability outcomes (Multimedia Appendix 2). The clinical psychologist will follow general prompts with questions and probes to develop a more comprehensive understanding of the participants’ perspectives.

### Data Analyses

Audio data will be transcribed verbatim using a transcription service and deidentified. Deidentified transcripts will be uploaded into Dedoose software [34] for formal analysis. We will use a combination of deductive and inductive approaches to data analysis for our key research aims. Specifically, our *deductive* approaches will serve as the basis for our interview guide, rapid data analysis template, and coding framework (ie, codebook domains), which will be influenced by (1) prior research on the dyads’ early psychosocial challenges and unmet needs following ADRD diagnoses and (2) the findings from on our prior studies focused on dyadic intervention development [35]. For example, we will include prompts in the interview guide that assess perspectives of the proposed delivery context (eg, current available psychosocial resources and ways of screening and referring dyads) on the rapid data analysis template and initial codebook domains.

Following the organization of the initial codebook domains, our approach to data analysis will involve rapid data analysis and be primarily *inductive* in nature. During data collection, our research assistant will document observations using the rapid data analysis template in a generative manner based on focus group discussion. These observations will be used to refine our initial codebook prior to formal data analysis and to assess when thematic saturation is reached [36]. Next, we will analyze the transcribed interviews in a collaborative and iterative fashion guided by the framework method [37]. Members of our research team involved in data analysis will begin with a line-by-line review of transcripts for 1-2 focus groups and openly code any relevant observations. These codes will be organized into categories within the a priori determined domains and refined through team discussion to create a revised codebook that will be used for formal coding in Dedoose. Two members of the research team will then code 25% (n=1-2 focus groups) of the transcripts using the initial codebook and will resolve discrepancies in discussions with the larger research team to refine the codebook. We will ensure a high agreement between the coders before they individually complete the remaining focus group transcriptions. We will then iteratively refine coded data into themes and subthemes within each domain through individual review and team discussions.

## Results

This study is funded by the National Institute on Aging grant 1K23AG075188-01A1. Recruitment began in November 2022. Data collection is anticipated to begin in January 2023 and be completed by June 2023, and data analysis is anticipated to be completed by August 2023.

## Discussion

ADRD are increasingly common conditions due to the aging population and have a considerable negative impact on persons living with dementia and their spousal care partners. At present, these dyads have little psychosocial support to address postdiagnosis challenges and positively adjust to ADRD and progressive symptoms at the time of diagnosis. Dyads are interested in participating in interventions together early after diagnosis [10-14], and prior research suggests that existing interventions [24,25] can be adapted to effectively and efficiently address the dyads’ challenges to promote dyadic adjustment [15,16]. To effectively adapt such programs, it is necessary to first gather comprehensive feedback from individuals who will adopt, deliver, and use interventions [14,27]. This approach can help promote alignment with patient, caregiver, and medical stakeholder perspectives and is recognized as an important step toward optimizing clinical care for ADRD [28,29].

This paper describes a study design that is part of the first phase of a larger project focused on the development of a novel postdiagnosis dyadic resiliency intervention for couple dyads navigating ADRD. We provide details on the study aims and the recruitment, screening, data collection, and data analysis procedures. This information is valuable for future studies involving medical stakeholders treating ADRD shortly following diagnosis and for the development of intervention programs and procedures that are tailored to address the postdiagnosis experience of persons living with dementia and their care partners.

The results of this trial will critically inform the development of the proposed RT-ADRD intervention and procedures. Our findings will also benefit the broader field, as we will gain perspectives to further understand common experiences and challenges before and after ADRD diagnoses. In addition, using direct feedback from a variety of stakeholders, we will be able to highlight commonly identified ways of integrating interventions within the existing clinical infrastructures, as well as areas of disagreement across stakeholder groups and clinic sites. Taken together with feedback obtained from dyads at the time of diagnosis gathered from a separate study, our findings will be used to refine our approach prior to pilot-testing, consistent with the National Institutes of Health Stage Model guidelines [31].

In summary, this study will be the first to gather comprehensive feedback from medical stakeholders on experiences, unmet needs, and perceptions of helpful intervention approaches and procedures for persons living with couple dyads early after ADRD diagnoses. Our findings will inform the design, procedures, and content of the novel Resilient Together for Dementia program, and we will gather feedback to inform subsequent clinical trials and implementation efforts. The results from this study will also have the potential to directly inform other efforts to increase the dyads’ postdiagnosis support, with implications for other chronic and progressive neurological conditions.

## Appendix

Multimedia Appendix 1 Rapid data analysis template.

Multimedia Appendix 2 Focus group guide.

Multimedia Appendix 3 Peer-review reports from the Career Development for Clinicians/Health Professionals Study Section - National Institute on Aging Initial Review Group - National Institute on Aging (National Institutes of Health, USA).

## Abbreviations

ADRD: Alzheimer disease and related dementias
RT-ADRD: Resilient Together for Alzheimer disease and related dementias

## Data Availability

Data collection and analysis instruments are available in the Multimedia Appendices. The data sets generated or analyzed during this study are available from the corresponding author on reasonable request.
