# Supplementary material for: Focus Group Study of Medical Stakeholders to Inform the Development of Resilient Together for Dementia: Protocol for a Postdiagnosis Live Video Dyadic Resiliency Intervention
Source: JMIR Res Protoc. 2023 May 29;12:e45533. doi: 10.2196/45533 (PMC10262018; doi:10.2196/45533)
Supplement: Multimedia Appendix 1 [file resprot_v12i1e45533_app1.docx]

**Multimedia Appendix 1: Rapid Data Analysis Template**

| **Date:**  **Prepared by:**  **Interviewer:** |
| --- |
| **Stakeholder Information:**  ***Occupation:***  ***Years of Experience Working with PWD:***  ***Other relevant characteristics*** *(who the person provides care to):* |
| **Domain 1: Clinical Care Early after ADRD Diagnosis** |
| **RT-D Intervention Recommendations and Procedures**  **General Impressions** |
| **Session Content** |
| **RT-D Procedures**  **Screening Procedures**  **Recruitment Procedures**  **Maximizing Feasibility and Acceptability Outcomes** |
| **Important observations and reflections**  ***Broad themes and topics of interest in the interview*** *(e.g., things not covered in rapid data analysis domains*)**:**  **Important quotations** |
| **Interviewee analytic notes + reflexivity** (e.g., any aspects of researchers’ identity, beliefs, social positioning and how they might influence the interview content and observations—consider how your field notes are your own *interpretations* of the interactions that took place)**::** |
| **Behind-the-scenes information (before or after recording; nonverbal information; etc.):** |
