# Supplementary material for: Focus Group Study of Medical Stakeholders to Inform the Development of Resilient Together for Dementia: Protocol for a Postdiagnosis Live Video Dyadic Resiliency Intervention
Source: JMIR Res Protoc. 2023 May 29;12:e45533. doi: 10.2196/45533 (PMC10262018; doi:10.2196/45533)
Supplement: Multimedia Appendix 2 [file resprot_v12i1e45533_app2.docx]

**Multimedia Appendix 2: Focus Group Guide**

Our team is planning to develop a novel program “Resilient Together for Dementia” designed to prevent distress and promote resiliency among couples early after a diagnosis of an Alzheimer’s disease or a related dementia (ADRD). This program will be a dyadic intervention, meaning that it will involve patients and their spousal caregivers simultaneously. We hope to deliver this program within the first month after diagnosis, as no programs currently exist that provide partners with support during this period. We have some ideas about the potential procedures, format, and content of this intervention based on our prior work, and we hope to learn from you during this study.

The interview topics were selected to help our team understand how we can best support your work and develop a program that addresses your patients’ needs after diagnosis. We would also like to understand how to best collaborate with medical teams to recruit and engage patients and caregivers in our program.

Ultimately, we hope that this program will become a part of standard post-diagnosis care.

The focus groups will be held on Zoom. Please see below for recommended Zoom guidelines:

- Sit in a private, quiet place if you can.
- Please use the Zoom “Mute microphone” function at the bottom left of the screen to mute yourself when you are not speaking.
- Utilize the Zoom “Chat” feature at the bottom middle of the screen to add feedback throughout the focus group session if someone else is speaking or we have moved on to a different topic.

We have included a list of potential topics below to give you an idea of what to expect to discuss during the focus group sessions.

**Topic: Clinical Care Early after ADRD Diagnosis**

Experience of delivering an ADRD diagnosis to patients and their caregivers

Who attends appointments

How information is delivered

Typical follow-up visits and support

Points of contact

Providers’ challenges delivering diagnosis or communicating with couples

Providing referrals to support groups, individual counseling, couples therapy

Emotional distress (i.e., elevated depression, anxiety, stress) in patients and spouses after diagnoses

How common within the period 1-2 months after diagnoses

Mental health resources available to patients

Ideal scenario for addressing mental health concerns after diagnoses

Are centralized resources available for common challenges that patients and caregivers experience immediately (within 1-2 months) after diagnosis? If not, does your clinic typically provide resources for the following:

Emotional support

Understanding the diagnosis and next steps

Resources for help managing symptoms and adjusting to care-partnership

Communicating about difficult emotions and relationship changes

Planning for the future

**Topic: Intervention Recommendations and Procedures**

We are working to develop a program to support couples early after ADRD diagnoses. This program would be designed to help the couple communicate their own needs, emotions, and challenges, how to access support, whether and how to share the diagnosis, how to navigate challenges such as finances, legal issues, and planning for long term care.

**General Impressions**

What are your first impressions of a program like this?

What would you want this program to address?

What do you think couples would benefit most from learning?

**Referral Procedures**

In your opinion, what are the best practices for our study to be introduced to patients?

During clinic visit

Particular days staff are in-clinic

Person making initial contact

Method of contact (flyer from treating providers)

Logistical resources needed

Familiarizing staff with study (information needed)

Are there any barriers to recruitment that we should be aware of?

Staff/study team methods of identifying appropriate patients

Staff time constraints

Staff familiarity with eligibility and screening procedures

Others

It is of specific importance for our study staff to gather perspectives from a diverse sample of couples in terms of race and ethnicity. Can you think of any barriers to our team achieving this goal? Any recommended solutions?

**Screening Procedures**

What factors do you think are important for our team to assess to determine whether couples would be a good fit for an early dyadic intervention?

ADRD cognitive, emotional, behavioral symptoms

Comfort using virtual technology

Specific emotional concerns or distress

Relationship distress

In your opinion, are there any ADRD symptoms that would make couples NOT suitable for the program?

**Recruitment Procedures**

What do you think would serve as motivators for couples to participate?

In thinking recruiting couples for interviews and the intervention component of our research study, what comes to mind in terms of barriers that our team would need to overcome?

What are your general impressions of our recruitment materials?

**Maximizing Feasibility and Acceptability Outcomes**

How can we best build collaborations with clinical staff and our study team?

What is the best way to collect self-report measures from persons with dementia?

How can we maximize participants’ completion of self-report measures during our initial interviews and in the open pilot phase of the project (baseline and 6 weeks post-intervention)?

How can we maximize recruitment and retention of couples after ADRD diagnoses?
