# Supplementary material for: Focus Group Study of Medical Stakeholders to Inform the Development of Resilient Together for Dementia: Protocol for a Postdiagnosis Live Video Dyadic Resiliency Intervention
Source: JMIR Res Protoc. 2023 May 29;12:e45533. doi: 10.2196/45533 (PMC10262018; doi:10.2196/45533)
Supplement: Multimedia Appendix 3 [file resprot_v12i1e45533_app3.pdf]

SUMMARY STATEMENT
(Privileged Communication)

PROGRAM CONTACT:
Dr Elizabeth Necka
301-496-3136
liz.necka@nih.gov

Release Date: 02/22/2022
Revised Date:

Application Number: 1 K23 AG075188-01A1
Formerly: 1K23AG075188-01

Principal Investigator

BANNON, SARAH

Applicant Organization: MASSACHUSETTS GENERAL HOSPITAL

Review Group: AGCD-3
Career Development for Clinicians/Health Professionals Study Section

Meeting Date: 01/18/2022
Council: MAY 2022
Requested Start: 07/01/2022
RFA/PA: PA20-206
PCC: 2BBCHEN
Dual IC(s): NR

Project Title: Resilient Together for Dementia: A live video resiliency dyadic intervention for persons with dementia and their care-partners early after diagnosis
SRG Action: Impact Score:20
Next Steps: Visit https://grants.nih.gov/grants/next_steps.htm
Human Subjects: 30-Human subjects involved - Certified, no SRG concerns
Animal Subjects: 10-No live vertebrate animals involved for competing appl.
Gender: 1A-Both genders, scientifically acceptable
Minority: 1A-Minorities and non-minorities, scientifically acceptable
Age: 3A-No children included, scientifically acceptable

Project Year	Direct Costs Requested	Estimated Total Cost
1	181,912	196,186
2	181,906	196,180
3	181,956	196,234
4	181,995	196,276
5	181,995	196,276
TOTAL	909,764	981,151

ADMINISTRATIVE BUDGET NOTE: The budget shown is the requested budget and has not been adjusted to reflect any recommendations made by reviewers. If an award is planned, the costs will be calculated by Institute grants management staff based on the recommendations outlined below in the COMMITTEE BUDGET RECOMMENDATIONS section.

RESUME AND SUMMARY OF DISCUSSION: This resubmission application for a Mentored Patient-Oriented Research Career Development Award (Parent K23, Independent Clinical Trial Required) by the Massachusetts General Hospital (MGH), Boston, MA, on behalf of Dr. Sarah Bannon, requests five years of support to transition the candidate to an independent clinical research career focusing on early interventions for dyads of persons with dementia (PWDs) and their informal care-partners. The resubmitted application is mostly responsive to the criticisms raised in the previous evaluation. The candidate is well trained and experienced; she holds an MA and a PhD both in Clinical Psychology and obtained at the Stony Brook University; she is enrolled in a postdoctoral fellowship in Integrated Brain Health Clinical and Research at MGH/Harvard Medical School (HMS). Dr. Bannon is a productive scientist; she has authored approximately 30 publications (from the biosketch), some as first author (~16) and some also relevant to the theme of the application; about eight articles have been published after the initial submission. She has been the recipient of a few awards, independent funding (not in the field of the application) and professional recognitions. The career development plan (CDP) has been reworked and strengthened in response to the feedback; it is well organized, focused on strengthening the candidate's relative weakness, well aligned with the research goals, and includes a good mix of coursework, workshops, interaction with mentors, and hands-on trainings. The CDP also includes a sound individual development plan (IDP), appropriate timelines, and greatly informative tables and diagrams. The research plan (RP) is well framed in the scientific field of knowledge, innovative, interesting, and well crafted. It addresses an area of great need and innovatively combines mixed qualitative and quantitative methods research to develop and test a unique psychological treatment program for dyads of patients with ADRD and their spouse caregivers. The intervention is novel, includes the dispersion of needed video equipment, extended justifications have been provided in support of the pilot trial decision strategy, and availability of technical resources to the participants has been well addressed in the revised RP. The mentorship team includes crossdisciplinary experts that have excellent track records of publications, funding, and mentorship. It includes the primary mentor, Dr. Vranceanu (Associate Professor at MGH; clinical psychologist and expert in dyad intervention development); a co-mentor, Dr. Ritchie (Professor at MGH; geriatrician and expert in palliative care research; highly experienced mentor); and two advisors/collaborators, Drs. Dickerson (Associate Professor at HMS/MGH; neurologist, AD/ADRD, and imaging expert) and Cornelius (Assistant Professor at Columbia University; experienced biostatistician in clinical research). The institutional support provided in the letter of Dr. Fava, Director of the Division of Clinical Research at the MGH Research Institute, indicates a potential promotion and allows for 75%-protected research time. The environment at MGH/HMS is well suited to support the candidate's career development ambitions. Weaknesses of the application include the lack of whole-hearted institutional support, in fact, even though a promotion has been approved in summer of 2021, the applicant is still a postdoctoral fellow, the timeline for the advancement is not spelled out, and it appears that both promotion and protected research time are contingent on the award. Furthermore, the candidate's publication record was difficult to assess due to the discrepancies throughout the application and the outdated record in the link to MyBibliography. Health equity training remains superficial; some aspects of the RP are lacking details; the cultural underpinnings of delivering the intervention are not appropriately considered; and the content of the questionnaire lacks detailed descriptions. The mentorship team should be strengthened by the addition of a health equity specialist; in this respect the role of Dr. Jackson could be elevated/formalized to remediate this lingering weakness. In sum, the few and minor weaknesses slightly detract from the high enthusiasm for this application.

TRAINING IN THE RESPONSIBLE CONDUCT OF RESEARCH: Acceptable. The planned activities satisfy the requirement for training in the responsible conduct of research.

DESCRIPTION (provided by applicant): In this K23 proposal, I outline a comprehensive 5-year training program that will support my transition towards an independent investigator focused on the

development and rigorous testing of interventions for dyads (i.e., pairs) of persons living with dementia (PWDs) and their informal care-partners, with an emphasis on early intervention. In this application, I propose a significant and innovative proposal that is directly tied with my proposed training and career development goals. Background: Alzheimer's disease and related dementias (ADRD) produce a host of stressors for PWDs and their spousal care-partners (SPs), who both experience substantial emotional distress after diagnosis. Emotional distress is interdependent within dyads and – without treatment— becomes chronic and negatively impacts both partners' health, quality of life, and their ability to navigate the short and long-term challenges associated with ADRDs. Addressing emotional distress early, when PWDs can still meaningfully participate, is an unexplored opportunity to prevent chronic emotional distress and preserve quality of life for both partners. Specific aims and research design: I aim to develop the first version of the live video Resilient Together for Dementia (RT-D) intervention and methodology via 1) interviews and quantitative surveys (N=20) of PWD-SP dyads, with additional feedback from 2) focus groups with ADRD medical stakeholders (N=4) (Aim 1). Next, I will explore, via an open pilot (N=5 dyads) with exit interviews and pre-post self- report assessments, the initial feasibility, acceptability, and credibility of the live video RT-D and procedures, and to further refine RT-D as needed (Aim 2). Finally, I will establish, via a pilot feasibility RCT of the RT-D versus control (N= up to 50 dyads), the feasibility, acceptability and credibility of RT-D following predetermined benchmarks (Aim 3). Findings will inform a hybrid efficacy-effectiveness trial through the R01 mechanisms and future studies extending this work to include additional family members and other care-partners. Training and mentoring: My aims are supported by 3 training goals to develop expertise in: 1) qualitative and mixed methods assessment to inform intervention adaptation; 2) specialty training in geriatrics and ADRD clinical care; 3) clinical trial methodology to facilitate dyadic intervention development and refinement. I will obtain mentorship from an exemplary team led by my primary mentor Dr. Ana-Maria Vranceanu, a clinical health psychologist with expertise in mixed-methods research and live video dyadic intervention development, and my co-mentor Dr. Christine Ritchie, a geriatrician and palliative care physician with decades of work improving the treatment of ADRD. My training goals are supported by 1) a team of expert mentors, 2) a rich institutional environment at Massachusetts General Hospital and Harvard Medical School, and 3) targeted coursework, scientific meetings, seminars and planned publications. Relevance to the NIA mission. This K23 is in line with NIAs priorities to develop interventions to the maintain health and wellbeing and reduce the burden of ADRDs. Impact: I am a clinical psychologist with expertise in couple and family interventions for neurological populations. The experience gained will serve as the foundation for an independent career in dyadic interventions for ADRDs, with a focus on early intervention.

PUBLIC HEALTH RELEVANCE: The proposed study will establish the feasibility, acceptability and credibility of a novel live video dyadic resiliency intervention, Resilient Together for Dementia (RT-D), aimed at preventing chronic emotional distress and preserving quality of life among dyads at risk for chronic emotional distress early after a diagnosis of Alzheimer's disease or a related dementia (ADRD). Both persons living with dementia (PWDs) and their spousal care-partners experience high levels of clinically elevated emotional distress, which can become chronic without treatment and negatively impact the health, quality of life, communication, and care-planning of both partners. A tailored dyadic intervention delivered over live video to this at risk population during the window of opportunity when PWDs can participate has the potential to prevent chronic emotional distress and preserve quality of life for PWDs and their loved ones.

DISCLAIMER: Please note that the following critiques were prepared by the reviewers prior to the Study Section meeting and are provided in an essentially unedited form. While there is opportunity for the reviewers to update or revise their written evaluation, based upon the group’s discussion, there is no guarantee that individual critiques have been updated subsequent to the discussion at the meeting. Therefore, the critiques may not fully reflect the final opinions of the individual reviewers at the close of

group discussion or the final majority opinion of the group. Thus, the Resume and Summary of Discussion is the final word on what the reviewers actually considered critical at the meeting.

CRITIQUE 1:

Candidate:1
Career Development Plan/Career Goals /Plan to Provide Mentoring:1
Research Plan:3
Mentor(s), Co-Mentor(s), Consultant(s), Collaborator(s):1
Environment Commitment to the Candidate:1

Overall Impact:
This is a very strong resubmission K23 application by an exceptional candidate. The application is very well put together on a compelling, clinically relevant, and refreshingly novel topic in the world of ADRD. The candidate has excellent training in clinical psychology and has built an early career focusing on dyadic interventions. She has an impressive publication record, which has grown since the initial submission, notably in areas more related to Alzheimer's disease (AD) and related dementia (ADRD) topics. She has received highly competitive awards in the past. The CDP is organized, maps on well to career goals, is an appropriate mix of activities, and is focused appropriately on strengthening areas of relative weakness. The RP is very interesting. It combines mixed qualitative and quantitative methods innovatively to develop a unique psychological treatment program for patients with ADRD and their caregiver. This type of intervention, especially in the face of no available pharmacological interventions for ADRD that are particularly clinically useful, is sorely needed, yet, to this reviewer's knowledge, has not been the focus of previous efforts. The work leverages the candidate's experience and the expertise of her mentorship team, while simultaneously carving out a distinct scientific niche that will ultimately be attributed unambiguously to her work. The research lays out an appropriate iterative plan to develop and evaluate the intervention program. It is well conceptualized and clearly described. Concerns about this submission are relatively minor. Although some of the criticisms raised previously were minor, they were impactful, and the candidate could have done a more thorough and thoughtful job in responding to them. Most of the previous work on dyadic therapy has been done with patients with either "static" impairment or with great potential for cognitive improvement. There is concern that ADRD, defined by cognitive decline and dementia, will, quite simply, lead to forgetting of skills covered during the sessions or reduced ability to learn new skills. Of course, patients with ADRD maintain degrees of ability to learn and retain new information, but there is tremendous variability both between patients and within patients over time. It is unclear if the applicant has fully contemplated this issue and has an explicit plan with dealing with it. Moreover, it is unclear if simply mapping a successful dyadic program onto this unique population defined by neurodegeneration will work. Somewhat ironically, but almost by definition, the skills and strategies learned early in the disease process might be less accessible later in the disease process when patients are affected more cognitively, yet it is later in the disease process where implementation of dyadic strategies is probably most relevant. Research, such as the work proposed, is the obvious way to test these issues, but the concern is about whether there is existing evidence or appropriate explicit consideration in the application of the approach to this population over time. The candidate has published a review since the first submission that appears to make a case for dyadic intervention in this population, but the response in the application is somewhat superficial (e.g., "I will adjust for the unique challenges of ADRD…"). There is doubt that the cognitive and neurodegenerative course of ADRD will be the primary reason why this intervention will not be particularly affective, especially when compared with similar intervention strategies in other populations (the foundation for the work proposed) or approaches that focus exclusively on caregivers. Similarly, superficial were responses to concerns about cultural factors that may be quite relevant in the

intervention. The candidate promises to test difference across demographic groups to power a more definitive prospective study, but the issues is not really about “main effect” differences across demographic groups. Rather, it is about the incorporation of strategies in the intervention itself to make it accessible and appropriate for all cultural groups. The mentorship team is ideal to support the work and the institution/institutional commitment is outstanding.

1. Candidate:
Strengths
- Candidate has exceptional clinical training in relevant areas for this application.
- Similarly, she has great research training.
- She has been extremely productive, with evidence of a focused, iterative, burgeoning research program. Since the last submission she has authored and published several manuscripts, several with direct relevance to the themes presented in the application.
- She has been successful with previous awards, grant applications, and recognitions.

Weaknesses
- None noted.

2. Career Development Plan/Career Goals and Objectives:
Strengths
- The plan is well organized and consistent with the career goals.
- The plan combines structured coursework, workshops, interaction with mentors, and applied work as vehicles for training.
- Candidate used tables quite effectively, particularly with respect to training goals and the description of the skill, current skill level, and gap in training.
- Appropriate timeline.
- Candidate incorporated IDP into training plan.

Weaknesses
- None noted.

3. Research Plan:
Strengths
- Overall, a very innovative and exciting RP that addresses a clinical area of great need.
- Excellent implementation of mixed qualitative and quantitative methods to develop the program.
- The work is consistent with the candidate’s past work and leverages the expertise of her mentorship team.
- Video conferencing equipment will be provided to participants.

Weaknesses
- Incorporation of strategies to address individual differences in severity of cognitive symptoms and other key behaviors (insight, neuropsychiatric symptoms) somewhat superficial.
- Strategy to incorporate cultural aspects into the therapeutic approach is also somewhat superficial.
- Basic psychoeducation related to what dementia is, what to expect in the course, advanced directives, etc. should be incorporated into the therapeutic strategy.

4. Mentor(s), Co-Mentor(s), Consultant(s), Collaborator(s):
Strengths
- Exceptionally qualified mentoring team; no concerns.

Weaknesses
- None noted.

5. Environment:
Strengths
- The environment is ideal to support this work.

Weaknesses
- Dr. Fava's letter notes that department's Executive Committee on Promotions endorsed Dr. Bannon's advancement to Assistant Professor in June 2021 and that her current appointment is instructor. However, there is no indication of when this promotion will take place and Dr. Bannon's biosketch notes that she is postdoctoral fellow, raising concern that the promotion is contingent on the funding of this award.

Study Timeline:
Strengths
- Appropriate.

Weaknesses
- None noted.

Protections for Human Subjects:
Acceptable Risks and Adequate Protections.
- Appropriate.

Budget and Period of Support:
Recommend as Requested.

CRITIQUE 2:

Candidate:1
Career Development Plan/Career Goals/Plan to Provide Mentoring:2
Research Plan:1
Mentor(s), Co-Mentor(s), Consultant(s), Collaborator(s):1
Environment Commitment to the Candidate:1

Overall Impact:
This is a resubmission K23 application by Dr. Bannon, a post-doctoral fellow at MGH/HMS in the Department of Psychiatry. Overall, this resubmission is highly responsive to the prior critique, with modifications that have substantially strengthened all areas of the proposed career development award project. Dr. Bannon continues to be an excellent candidate with newly added additional scientific products in the direct area of research focus. She proposes a comprehensive 5-year training plan towards becoming an independent investigator in the area of development/testing of interventions for dyads of persons living with dementia and their care-partners. Training and mentoring focus on qualitative and mixed methods assessment to inform intervention adaptation, specialty clinical training in geriatrics and ADRD care, and training in clinical trials methodologies. The training plan has been modified in response to prior critique and has been considerably strengthened. RP includes development and refinement of the "Resilient Together for Dementia" intervention (live video), an extension of the mentor's work in care support interventions for non-dementia populations. Mixed methods and a pilot RCT for feasibility are all incorporated into the RP, which has been modified and strengthened in response to the prior critique. Mentors continue to be excellent. Institutional support is considerably strengthened on this resubmission. Overall, an excellent application that will provide a successful foundation towards a future independent research career for this candidate.

1. Candidate:
Strengths
- Highly productive candidate—14 manuscripts total noted in candidate statement; 30 noted in biosketch. Numbers differ in different places throughout the application. Regardless, the candidate is productive. It was possible to verify that the most recent publications, although not listed on My NCBI, are present on-line. At least two of these are verified as first author publications directly relevant to the dementia field since 2020.
- Clear dedication to a research career.
- Well trained and seems ideally positioned to embark on a career development training pathway.

Weaknesses
- Please be consistent in reporting of scientific products throughout.
- My NCBI weblink is not updated appropriately and only lists 9 manuscripts. Many publications listed on the biosketch are missing from the MyBibliography record. Please ensure MyBibliography record is updated.
- No pilot grants in targeted area.

2. Career Development Plan/Career Goals and Objectives:
Strengths
- Training and mentoring focus on qualitative and mixed methods assessment to inform intervention adaptation, specialty training in geriatrics and ADRD care, and training in clinical trial methodologies. The training plan has been modified in response to prior critique and has been considerably strengthened to be more in-depth and robust.
- Coursework, training, and experiential learning has been added in implementation science, geriatric medicine/ADRD, and some limited work in health equity.

Weaknesses
- Health equity training is superficial and could be enhanced. Formally adding Dr. Jackson, a noted health equity research expert, to this application as a mentor would have considerably strengthened the application. As it stands, he is mentioned briefly as a colleague of the primary mentor, but not otherwise formally included nor is a statement of commitment is offered from Dr. Jackson.

3. Research Plan:
Strengths
- The RP includes development and refinement of the “Resilient Together for Dementia” intervention (live video), an extension of the mentor’s work in care support interventions for non-dementia populations. Mixed methods and a pilot RCT for feasibility are all incorporated into the RP, which has been modified and strengthened in response to the prior critique.
- A clearer theoretical framework is now present.
- Primary care is included in the recruitment plan (not just neurology).
- Populations are more clearly characterized.
- Justification and appropriate limitations are offered for limiting the dyads to co-habiting spousal care partners.
- Social determinants of health and modifications for limitations (functional) of ADRD are considered, albeit in a limited fashion.

Weaknesses
- Content of the baseline questionnaires remains vague.
- Although unintended consequences could likely be mitigated by the planned study team, as noted in the application, they should still be characterized and recorded as it would be done in any interventional study.

4. Mentor(s), Co-Mentor(s), Consultant(s), Collaborator(s):
Strengths
- Mentors remain outstanding, well qualified, committed with clear roles identified.

Weaknesses
- As noted above, an in-depth health disparities/equity mentor would strengthen the application. The primary mentor is noted as moving into this area, but an established health equity mentor would offer additional needed knowledge, experience, and support.

5. Environment:
Strengths
- The institutional commitment to the candidate has been substantially increased since the last submission. On June 15, 2021, the candidate's advancement to Assistant Professor (investigator track) was approved for MGH.
- This is clearly a well-resourced environment.

Weaknesses
- None noted.

Study Timeline:
Strengths
- Adequate.

Weaknesses
- None noted.

Protections for Human Subjects:
Acceptable Risks and Adequate Protections.

Data and Safety Monitoring Plan:
Acceptable.

Inclusion Plans:
- Sex/Gender: Distribution justified scientifically.
- Race/Ethnicity: Distribution justified scientifically.
- Inclusion/Exclusion Based on Age: Distribution justified scientifically.

Training in the Responsible Conduct of Research:
Acceptable.

Resource Sharing Plans:
Acceptable.

Budget and Period of Support:
Recommend as Requested.

CRITIQUE 3:

Candidate:1
Career Development Plan/Career Goals/Plan to Provide Mentoring:1
Research Plan:2
Mentor(s), Co-Mentor(s), Consultant(s), Collaborator(s):1

Environment Commitment to the Candidate:2

Overall Impact:
This is a resubmission of a K23 application requesting 5 years of support from MGH on behalf of Dr. Bannon, a licensed clinical neuropsychologist and researcher in the Integrated Brain Health Clinical and Research Program. The applicant has been highly responsive to prior reviews, which has resulted in a robust CDP and even stronger RP. Additionally, the applicant is to be commended for productivity during the interim between submissions with 8 new publications in the ADRD literature, for a total of 30 peer-reviewed publications (16 as first author) and invited talks on dyadic research. Enthusiasm remains very high for this exceptional candidate focused on an important and under-studied aspect of ADRD need.

1. Candidate:
Strengths
- Exceptionally productive candidate on track for a strong national presence in dyadic research.
- First generation college student.
- Clinical neuropsychologist focused on a high-priority disease and an important clinical need (emotional support/resilience training for PWD and care partners at time of ADRD diagnosis) that her previous training prepares her well to address.

Weaknesses
- None noted.

2. Career Development Plan/Career Goals and Objectives:
Strengths
- The three goals are appropriate and specific and well-aligned with the RP and longer-term goals.
- The additional didactic activities and training experiences included were responsive to prior critiques and justify 5 years of training.
- Plans have now been included for the involvement of a mentor expert in Ethics and RCR training, and the promotion of diversity and equity in research training; the CDP now includes also an IDP.

Weaknesses
- None noted.

3. Research Plan:
Strengths
- The details that were lacking or under-specified in the prior submission have now been clarified.
- Justification is provided for main decision points in selection of measures and target population for this pilot work.
- The approach applies the Stage Model to progress through intervention development, open pilot, and feasibility pilot RCT.
- New approaches have been included and appropriately budgeted to address internet accessibility and comfort level.

Weaknesses
- Minimally enhanced usual care, the control condition for Aim 3, is not specifically described.
- Even though it is reasonable to conduct this pilot work in a single academic center setting in Boston, it will be critical to plan for more generalizable adaptation and evaluation of this intervention in future studies, by expanding to additional sites and reaching more diverse study populations.

4. Mentor(s), Co-Mentor(s), Consultant(s), Collaborator(s):
Strengths
- This is a strong and well-rounded mentorship team.

Weaknesses
- None noted.

5. Environment:
Strengths
- Outstanding environment to support this project.
- She has been appointed to faculty and the candidate's department has voted for promotion to Assistant Professor.

Weaknesses
- Protection of 75% time still seems to be contingent on receipt of this award.

Protections for Human Subjects:
Acceptable Risks and Adequate Protections.

Data and Safety Monitoring Plan:
Acceptable.

Inclusion Plans:
- Sex/Gender: Distribution justified scientifically.
- Race/Ethnicity: Distribution justified scientifically.
- Inclusion/Exclusion Based on Age: Distribution justified scientifically.

Resubmission:
- Highly responsive.

Training in the Responsible Conduct of Research:
Acceptable.

Resource Sharing Plans:
Acceptable.

Budget and Period of Support:
Recommend as Requested.

THE FOLLOWING SECTIONS WERE PREPARED BY THE SCIENTIFIC REVIEW OFFICER TO SUMMARIZE THE OUTCOME OF DISCUSSIONS OF THE REVIEW COMMITTEE, OR REVIEWERS' WRITTEN CRITIQUES, ON THE FOLLOWING ISSUES:

PROTECTION OF HUMAN SUBJECTS: ACCEPTABLE. No concerns were reported by the scientific review group pertaining to the compliance to NIH regulations for the protection of human subjects.

INCLUSION OF WOMEN PLAN: ACCEPTABLE. No concerns were reported by the scientific review group regarding the enrollment of women.

INCLUSION OF MINORITIES PLAN: ACCEPTABLE. No concerns were reported by the scientific review group in relation to minority groups' recruitment.

INCLUSION ACROSS THE LIFESPAN: ACCEPTABLE. No concerns were reported by the scientific review group regarding the inclusion throughout the lifespan; children exclusion is scientifically justified.

COMMITTEE BUDGET RECOMMENDATIONS: The budget was recommended as requested.

Footnotes for 1 K23 AG075188-01A1; PI Name: Bannon, Sarah

NIH has modified its policy regarding the receipt of resubmissions (amended applications).See Guide Notice NOT-OD-18-197 at https://grants.nih.gov/grants/guide/notice-files/NOT-OD-18-197.html. The impact/priority score is calculated after discussion of an application by averaging the overall scores (1-9) given by all voting reviewers on the committee and multiplying by 10. The criterion scores are submitted prior to the meeting by the individual reviewers assigned to an application, and are not discussed specifically at the review meeting or calculated into the overall impact score. Some applications also receive a percentile ranking. For details on the review process, see http://grants.nih.gov/grants/peer_review_process.htm#scoring.

MEETING ROSTER

Career Development for Clinicians/Health Professionals Study Section
National Institute on Aging Initial Review Group
NATIONAL INSTITUTE ON AGING
AGCD-3
01/18/2022 - 01/19/2022

Notice of NIH Policy to All Applicants: Meeting rosters are provided for information purposes only. Applicant investigators and institutional officials must not communicate directly with study section members about an application before or after the review. Failure to observe this policy will create a serious breach of integrity in the peer review process, and may lead to actions outlined in NOT-OD-14-073 at https://grants.nih.gov/grants/guide/notice-files/NOT-OD-14-073.html, NOT-OD-15-106 at https://grants.nih.gov/grants/guide/notice-files/NOT-OD-15-106.html, and NOT-OD-18-115 at https://grants.nih.gov/grants/guide/notice-files/NOT-OD-18-115.html, including removal of the application from immediate review.

CHAIRPERSON(S)

KELLEY, AMY STEVES, MD
PROFESSOR
BROOKDALE DEPARTMENT OF GERIATRICS AND PALLIATIVE MEDICINE
ICAHN SCHOOL OF MEDICINE AT MOUNT SINAI
NEW YORK, NY 10029

VIDAL, RUBEN, PHD
PROFESSOR
DEPARTMENT OF PATHOLOGY AND LABORATORY MEDICINE
SCHOOL OF MEDICINE
INDIANA UNIVERSITY
INDIANAPOLIS, IN 46202

MEMBERS

ANTON, STEPHEN D, PHD, MS, BS *
ASSOCIATE PROFESSOR
DEPARTMENT OF AGING AND GERIATRICS
CHIEF
DIVISION OF CLINICAL RESEARCH
UNIVERSITY OF FLORIDA, COLLEGE OF MEDICINE
GAINESVILLE, FL 32611

BRICKMAN, ADAM M., PHD
PROFESSOR
TAUB INSTITUTE FOR RESEARCH ON ALZHEIMER'S DISEASE AND THE AGING BRAIN
DEPARTMENT OF NEUROLOGY
COLUMBIA UNIVERSITY
NEW YORK, NY 10027

CHIN, JEANNIE, PHD *
ASSOCIATE PROFESSOR
DEPARTMENT OF NEUROSCIENCE
MEMORY AND BRAIN RESEARCH CENTER
BAYLOR COLLEGE OF MEDICINE
HOUSTON, TX 77030

KIND, AMY J., MD, PHD
PROFESSOR & DIRECTOR
CENTER FOR HEALTH DISPARITIES RESEARCH
SCHOOL OF MEDICINE AND PUBLIC HEALTH
UNIVERSITY OF WISCONSIN
MADISON, WI 53705

KOROUKIAN, SIRAN M., PHD
PROFESSOR
DEPARTMENT OF POPULATION AND QUANTITATIVE HEALTH SCIENCES
CASE WESTERN RESERVE UNIVERSITY
CLEVELAND, OH 44106

LEW, SUSIE Q., MD *
PROFESSOR
DEPARTMENT OF MEDICINE
GEORGE WASHINGTON UNIVERSITY MEDICAL CENTER
WASHINGTON, DC 20037

MACK, WENDY JEAN, PHD
PROFESSOR
DEPARTMENT OF POPULATION AND PUBLIC HEALTH SCIENCE
UNIVERSITY OF SOUTHERN CALIFORNIA
LOS ANGELES, CA 90033

MANNING, CAROL A, BA, PHD *
PROFESSOR
PROFESSOR AND DIRECTOR MEMORY DISORDERS CLINIC
DEPARTMENT OF NEUROLOGY
UNIVERSITY OF VIRGINIA
CHARLOTTESVILLE, VA 22908

MARSHALL, GAD ASHER, MD
ASSOCIATE PROFESSOR
DEPARTMENT OF NEUROLOGY AND
CENTER FOR ALZHEIMER RESEARCH & TREATMENT
BRIGHAM AND WOMEN'S HOSPITAL
BOSTON, MA 02115

MORENO, GERARDO, MD, MS
ASSOCIATE PROFESSOR
DEPARTMENT OF FAMILY MEDICINE
DAVID DEFFEN SCHOOL OF MEDICINE
UNIVERSITY OF CALIFORNIA, LOS ANGELES
LOS ANGELES, CA 90024

OH, ESTHER SEUNGHEE, MD, PHD *
ASSOCIATE PROFESSOR
DEPARTMENT OF PATHOLOGY
DIVISION OF NEUROPATHOLOGY
JOHNS HOPKINS UNIVERSITY SCHOOL OF MEDICINE
BALTIMORE , MD 21224

PROMISLOW, DANIEL EDWARD, PHD
PROFESSOR
DEPARTMENT OF LAB MEDICINE & PATHOLOGY AND
DEPARTMENT OF BIOLOGY
UNIVERSITY OF WASHINGTON
SEATTLE, WA 98195

WHITSON, HEATHER E., MD, HEALTH SCI *
ASSOCIATE PROFESSOR OF MEDICINE
DEPARTMENT OF MEDICINE
DUKE UNIVERSITY
DURHAM, NC 27708

WOLK, DAVID A., MD
PROFESSOR
DEPARTMENT OF NEUROLOGY
UNIVERSITY OF PENNSYLVANIA
PHILADELPHIA, PA 19104

SCIENTIFIC REVIEW OFFICER

GRIMALDI, MAURIZIO, PHD, MD
SCIENTIFIC REVIEW OFFICER
NATIONAL INSTITUTE ON AGING
NATIONAL INSTITUTES OF HEALTH
BETHESDA, MD 20892

EXTRAMURAL SUPPORT ASSISTANT

NUCCI, JAMES BALDWIN
EXTRAMURAL SUPPORT ASSISTANT
SCIENTIFIC REVIEW BRANCH
NATIONAL INSTITUTE ON AGING
NATIONAL INSTITUTES OF HEALTH
BETHESDA, MD 20892

* Temporary Member. For grant applications, temporary members may participate in the entire meeting or may review only selected applications as needed.

Consultants are required to absent themselves from the room during the review of any application if their presence would constitute or appear to constitute a conflict of interest.
